# p53-Related Transcription Targets of TAp73 in Cancer Cells—Bona Fide or Distorted Reality?

**DOI:** 10.3390/ijms21041346

**Published:** 2020-02-17

**Authors:** Chao Wang, Cui Rong Teo, Kanaga Sabapathy

**Affiliations:** 1 Division of Cellular and Molecular Research, Humphrey Oei Institute of Cancer Research, National Cancer Centre Singapore, Singapore 169610, Singapore; wang.chao@nccs.com.sg; 2 Cancer and Stem Cell Biology Program, Duke-NUS Medical School, Singapore 169857, Singapore; cuirong@u.duke.nus.edu; 3 Institute of Molecular and Cell Biology, Biopolis, Singapore 138673, Singapore; 4 Department of Biochemistry, Yong Loo Lin School of Medicine, National University of Singapore, Singapore 119228, Singapore

**Keywords:** Cancer cell lines, p53, TAp73, transcriptional targets, tumor suppression

## Abstract

Identification of p73 as a structural homolog of p53 fueled early studies aimed at determining if it was capable of performing p53-like functions. This led to a conundrum as *p73* was discovered to be hardly mutated in cancers, and yet, TAp73, the full-length form, was found capable of performing p53-like functions, including transactivation of many p53 target genes in cancer cell lines. Generation of mice lacking p73/TAp73 revealed a plethora of developmental defects, with very limited spontaneous tumors arising only at a later stage. Concurrently, novel TAp73 target genes involved in cellular growth promotion that are not regulated by p53 were identified, mooting the possibility that TAp73 may have diametrically opposite functions to p53 in tumorigenesis. We have therefore comprehensively evaluated the TAp73 target genes identified and validated in human cancer cell lines, to examine their contextual relevance. Data from focused studies aimed at appraising if p53 targets are also regulated by TAp73—often by TAp73 overexpression in cell lines with non-functional p53—were affirmative. However, genome-wide and phenotype-based studies led to the identification of TAp73-regulated genes involved in cellular survival and thus, tumor promotion. Our analyses therefore suggest that TAp73 may not necessarily be p53’s natural substitute in enforcing tumor suppression. It has likely evolved to perform unique functions in regulating developmental processes and promoting cellular growth through entirely different sets of target genes that are not common to, and cannot be substituted by p53. The p53-related targets initially reported to be regulated by TAp73 may therefore represent an experimental possibility rather than the reality.

## 1. TAp73—The Structural Homolog of p53, but a True Functional Homolog as Well?

p73, a member of the p53 family of transcription factors, shares high sequence homology with p53 at the amino acid level, being at least 29% similar in sequence identity in the N-terminal transactivation domain (TAD), 63% in the central DNA binding domain (DBD), and 38% in the oligomerization domain [1,2]. Its two major N-terminal isoforms are either with (TAp73) or without (DNp73) the TAD. Given the presence of a similar TAD and DBD in TAp73, it has been thought to perform functions akin to p53, which is an undisputed tumor suppressor that is mutated in over 50% of all cancers, or with its activity often being compromised in the others [3,4,5,6,7]. It has been well established that p53 inhibits cell growth upon DNA and cellular damage, by arresting the cell cycle through the transactivation of *p21*, by initiating apoptosis by transactivating *Puma*, or by triggering senescence through the transactivation of *PML* [5,8], among other target genes. Similarly, ectopic expression of TAp73 was shown to enhance cell death in many cancer cell lines, through TAp73-mediated transactivation of pro-apoptotic genes such as *Puma*, *Noxa*, and *Bim*, and thus inhibited cellular growth [9,10,11,12,13]. Moreover, TAp73 was also found to transcriptionally induce canonical p53 target genes involved in cell cycle arrest, such as *p21* [14], contributing to growth arrest. 

These early studies collectively demonstrated the ability of TAp73 to bind to the p53-response elements (p53RE) in canonical p53-target gene promoters and transactivate them, suggesting an overlapping role for TAp73 in contributing to growth inhibition. However, it is unclear whether growth inhibition and thus tumor suppression is indeed the primary function of TAp73, for a few reasons. Firstly, unlike p53, TAp73 is rarely mutated but has been suggested to be overexpressed in a variety of cancers such as breast cancer [15,16], bladder cancer [17], prostate cancer [18], and colorectal cancer [19], amongst others [20,21], alluding to the possibility of a diametrically opposite function to tumor suppression. Next, while hundreds of direct p53 target genes have been identified and functionally validated [22], TAp73 target genes are continually being discovered. Of those, only a subset includes the canonical p53 target genes involved in limiting cellular growth, whereas a significant portion are either involved in regulating developmental phenotypes, or are non-canonical genes involved in promoting cellular growth and metastasis in a context-dependent manner. This latter category includes a variety of anti-apoptotic and pro-survival genes regulated by TAp73, including the anti-apoptotic *Caspase 2S* [23], the cell proliferation regulators *cyclin D1*, *CDC25c*, and *CDK1* [24,25,26], metabolism regulators *G6PD* and *PFKL* [27,28], and the pro-angiogenic *Vegf-A* [29], suggesting a pro-tumorigenic function for TAp73, thereby questioning TAp73’s role in tumor suppression. 

Analyses of mice deficient for p73 (without both TAp73 and DNp73), or specifically TAp73, have shown that they exhibit a myriad of developmental defects, such as neurodegeneration and infertility, and have compromised longevity [30,31]. Interestingly, though p53-deficient embryos do also display neuronal defects such as exencephaly and also have fertility issues [32,33], most of the p53-null mice die early, between 3 and 6 months of age, primarily due to the development of spontaneous tumors [34,35]. By contrast, absence of TAp73 does not lead to early onset of spontaneous tumors. These mice instead develop tumors significantly later in life after 18 months of age [31], further suggesting that TAp73’s primary function might not be in tumor suppression. 

We have therefore analyzed all TAp73 target genes reported in human cancer cell lines, to comprehensively evaluate their potential roles in regulating cellular growth. TAp73 target genes identified in regulating a variety of developmental phenotypes have been reviewed elsewhere [36,37], and thus, are not discussed here. We hope that this systematic review will shed some clarity on the state of affairs of TAp73 targets identified thus far and propose how the field could evaluate TAp73’s role in tumorigenesis with an open mind-set.

## 2. Analysis Methodology

In this review, we define TAp73 targets as genes where the direct binding of TAp73 to the promoters or regulatory regions has been reported, and whose expression has been validated to be regulated by TAp73. Target genes identified from high-throughput studies have been selected for analysis in this review only when they meet the above criteria, though thousands of unverified genes have been documented in many studies using genome-wide microarray or RNA-sequence analyses. Since this review mainly focuses on human cancers, studies investigating other species (e.g., mice, zebra fish) have not been included for analysis. 

**Figure 1:**
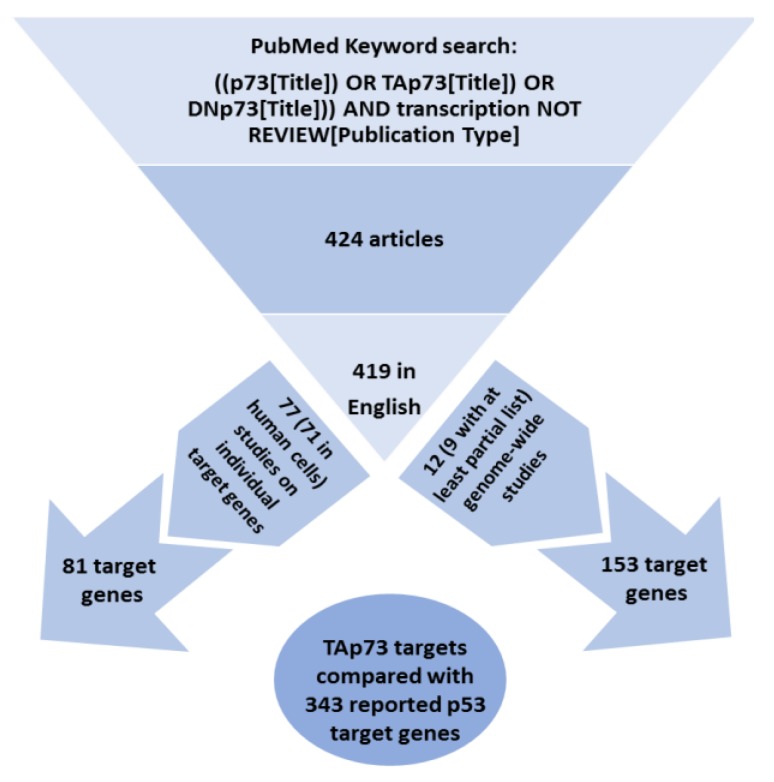
Overview of analysis. The flow diagram depicts the criteria used for the selection of articles reviewed in this report.

We performed a keyword search using the Boolean ((p73[Title]) OR (TAp73 [Title]) OR (DNp73 [Title])) AND transcription NOT REVIEW [Publication Type] in PubMed on 26 August 2019. The returned articles were further filtered by language to include English-based results only. From those articles, publications that met the above criteria for TAp73 target genes were considered relevant to the purpose of this review and were further grouped either as individual gene studies or genome-wide studies. Figure 1 illustrates the flow diagram of our analysis methodology.

We would like to highlight that studies describing other TAp73 transcriptional targets may have not been included in this analysis due to the nature of the search terms used for article identification. Nonetheless, we believe that the analysis provide a general overview that is representative of the TAp73 targets identified thus far. 

## 3. Targets Identified by Focused Studies—Overlapping with p53 Target Genes

Since p73 was first identified as a structural and functional homolog of p53 [38,39], its function as a tumor suppressor and a transcription factor has been intensely studied. In addition to its biochemical characterization and upstream regulation, a large number of target genes of p73 have been identified by individual studies or by high-throughput array-based screening. Our literature search using the keywords described above led to the retrieval of 424 articles. Five were not written in the English language and were excluded. The remaining 419 articles, published between 1997 and 2019, were independently reviewed by two authors. Among these articles, 77 were focused on one or a few individual target genes (Supplementary Table S1), and 12 were genome-wide analyses of p73 targets in human cancer cells (Supplementary Table S2). The remaining 300+ articles did not directly deal with TAp73 transcriptional targets but were investigating various aspects of p73 regulation, activity, and functions. 

**Figure 2:**
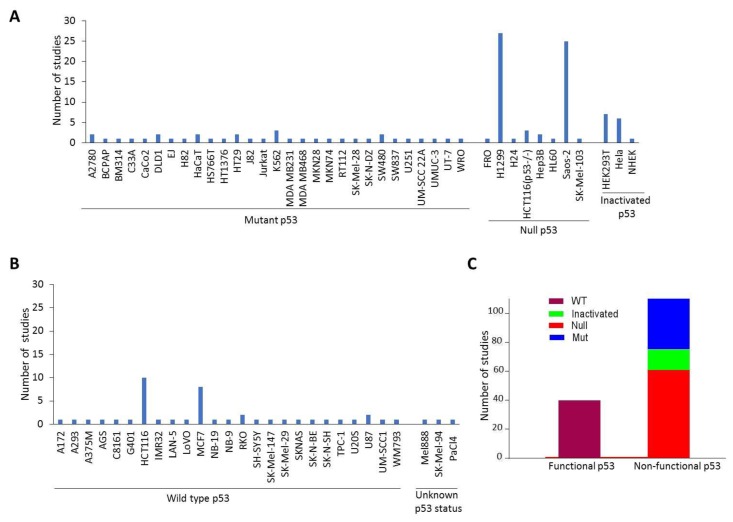
Cell lines used in identification of TAp73 targets. The frequency of cancer cell lines used in the 71 focused studies for identifying TAp73 target genes is depicted. (**A**) Cell lines with non-functional p53 and (**B**) cell lines with functional p53 or of unknown p53 status. (**C**) The number of studies using the various cell lines based on their p53 status is depicted. Please note that some studies used multiple cell lines.

Among the 77 focused articles, 6 solely utilized TAp73 knock-out mouse cells or other murine cell lines, and the remaining 71 studies were performed using human cell lines. In total, 68 different human cancer cell lines were employed in the 71 individual studies (Figure 2). The p53 status of the cancer cell lines used were recorded from the studies if described. In other cases, the p53 status was determined based on information obtained from online databases (e.g., www.p53.iarc.fr). More than half were without a functional p53: 8 cell lines are devoid of p53 expression (i.e., null) and 29 expressed mutant p53, and three cell lines, namely, HEK293T which was used in 7 studies, NHEK in 1 study, and Hela in 6 studies, are considered functionally inactive for p53 due to the overexpression of SV40 T antigen or genes of human papilloma virus (Figure 2A,B). While 3 cell lines carry p53 with unknown status, the remaining 25 cell lines expressed wild-type p53. The most utilized cell lines across these studies were the lung non-small cell carcinoma cell line H1299 and the osteosarcoma cell line Saos2, both null for p53 expression. H1229 was used 27 times, and Saos2 was used 25 times. In total, the number of studies using cell lines with functional wild-type p53 versus non-functional p53 was 43 versus 111, as presented in Figure 2C. 

An interesting feature of the studies in this category is that most of the experiments were conducted by ectopic overexpression of TAp73. A handful of studies utilized various stimuli known to induce TAp73 expression, including DNA-damaging conditions such as irradiation, etoposide, doxorubicin, and cisplatin treatment [28,40,41,42,43], or tumor-promoting conditions such as hypoxia [29,44,45,46], tumor necrosis factor (TNFα) treatment [47], and serum stimulation [48]. 

The target genes derived from the 71 individual focused studies that met our criteria are listed in Table 1. A total of 81 genes were identified as TAp73 targets, with the majority of them reported once in single studies. A few others were frequently reported in multiple studies. Of these, *CDKN1A (p21)*, *MDM2*, and *BAX* were the top 3 genes reported in multiple studies: 19, 12, and 12 studies respectively, as they were often used as positive controls. Firefly luciferase-based promoter assay was the most common method used to validate the regulation of target gene transcription upon TAp73 overexpression. The potential TAp73 binding sites were explored mainly by searching for typical p53REs in the proximal promoter regions, with the assumption that the binding sites would be evolutionally conserved and thus similar for TAp73 and p53. The majority of the 81 target genes contain a predicted p53RE in their genomic regulatory regions, of which 33 were validated.

When these TAp73 target genes were pooled together for Gene Ontology (GO) term analysis using the online gene set analysis tool Enrichr (https://amp.pharm.mssm.edu/Enrichr/), signaling pathways that emerged were DNA damage response, cell cycle arrest, and p53/p63/p73 networks (Table 2), similar to those that are activated by p53. Comparable results were also obtained using the online David gene set analysis tool (https://david.ncifcrf.gov/) (data not shown). It is noteworthy that many of these studies were aimed at identifying the target genes with intent, i.e., known p53 targets were evaluated to determine if they were also regulated by TAp73. Therefore, it is not surprising that the emerged pathways are similar to p53-regulated pathways and that these target genes are similarly regulated by p53. 

Comparing these 81 validated TAp73 target genes with the 343 validated p53 target genes identified from 319 individual gene studies [22], only 14 genes were found to be common, including *FAS*, *CCNG1*, *GLS2*, *CD82*, *SERPINB5*, *MDM2*, *PMAIP1*, *CDKN1A*, *PML*, *GDF15*, *BBC3*, *SFN*, *TP53INP1*, and *VDR.* Given the high sequence homology of TAp73 and p53, it was initially surprising that only a limited number of direct target genes are common between these two transcription factors. However, since the overlap of p53 target genes in various organs of the same animal is minimal [111], these data suggest that the contextual settings such as cell lines used and stimuli may contribute to the low overlap between p53 and TAp73 targets. Nevertheless, analysis of the 14 overlapping genes for biological processes and signaling pathways again showed that DNA damage response, cell cycle arrest, and apoptosis were top pathways (data not shown), similar to those obtained using the 81 TAp73 validated target genes. 

Altogether, the individual studies were generally designed to identify TAp73 target genes that are similar to p53 targets, in conditions where TAp73 is overexpressed and often in the absence of functional p53. The experimental set-up of these studies therefore reveals that the identified target genes can be regulated by TAp73 in the appropriate context, and hence can have overlapping functions with p53. However, whether these targets are bona-fide TAp73 targets capable of contributing to tumor suppression by TAp73 has remained an enigma.

## 4. Targets Identified from Genome-Wide Studies—Similar to and Different from p53 Target Genes 

In contrast to individual studies that identified and validated one or a few genes, high-throughput techniques such as microarray and Next-Generation Sequencing have also been used to identify TAp73 target genes across the entire genome, mostly focusing on coding genes. Based on our search term and analysis criteria, 12 articles published between 2008 and 2018 reported genome-wide studies aimed at identifying genes regulated by TAp73 in cancer cells [42,45,91,103,112,113,114,115,116,117,118,119]. These studies utilized various techniques such as microarray (six studies), RNA-Seq (one study), ChIP-on-ChIP (three studies), and ChIP-Seq (three studies). One study employed both RNA-Seq and ChIP-Seq [112], and another two studies have used either both the microarray and ChIP-Seq or the microarray and ChIP-on-ChIP [113,114] (Table 3). 

Similar to the individual studies, genome-wide analyses were carried out with different cell lines under different experimental conditions. In total, 11 cell lines were used in the 12 genome-wide studies, with the H1299 cell line being used twice. Nine out of the eleven cell lines are either null for p53 or express a mutated or inactivated/non-functional p53. The remaining two isogenic colon cancer cell lines, HCT116 and HCT116-3(6), contained wild-type p53 but differed in the expression of the MLH1 gene that was shown to be necessary for TAp73 activity [120,121].

Among these studies, two analyzed the effects of endogenous TAp73 on promoter binding upon TNF-α [115] or hydroxyurea treatment [116]. Five studies employed gene silencing using short-interfering (si)- or short-hairpin (sh)-RNAs against p73, among which, three studies evaluated the effects of cisplatin, rapamycin, and hydrogen peroxide (Table 3). Two other studies overexpressed TAp73α or TAp73β, and one used an inducible cell line to overexpress TAp73α or TAp73β. 

Nine out of the twelve articles provided a full or partial list of genes identified and included data on the validation of the targets. Incidentally, none of the target genes identified were common across all nine studies (Supplementary Table S2), likely reflecting the varied usage of cell lines and treatment conditions, as well as the potentially diverse roles that TAp73 could play in cancer cells under different contexts. Not unexpectedly, when we consolidated the signaling pathways that were provided in these genome-wide studies, no common signaling pathways emerged (data not shown). The lack of overlap could also be due to non-exhaustive gene lists that are publicly available, as well as potential false-positives associated with high-throughput screening assays, particularly in cases where ectopically overexpressed TAp73 may return a larger dataset compared to endogenous TAp73 targets. Furthermore, the ChIP-on-ChIP/ChIP-Seq techniques are highly dependent on the quality of antibodies used and sensitivity for chromatin immunoprecipitation. Four antibodies were used in six genome-wide ChIP studies: two home-made antibodies including #827 [116] and 11C12 [119], and two commercially available antibodies BL906 (Abcam) and NBP2-24737 (Novus Biologicals). While the p73 antibodies have been evaluated to recognize ectopically overexpressed TAp73 isoforms [122], none have been evaluated comprehensively for the detection of endogenous TAp73, specifically with the use of relevant knock-down/knock-out negative controls. 

Nevertheless, these genome-wide studies revealed hundreds to thousands of potential TAp73-regulatable genes. Of these, a portion had been validated. We chose 162 of these candidate genes that have been validated and demonstrated to be bound by TAp73 for analyses in this review (Supplementary Table S2). While no genes were common across all these high-throughput studies as mentioned before, genes such as *APOC3*, *C11orf21*, *GZMA*, *RPS27*, and *RRAD* were identified twice in separate studies, and genes such as *CDKN1A (p21)* and *MDM2* were identified thrice (Table 3), resulting in a total of 153 TAp73 target genes that could be verified from these genome-wide studies. GO analyses of the 153 TAp73 target genes identified signaling pathways involved in the p53 network and responses to genetic and oxidative stress, as well as activator-protein (AP)-1-related survival pathways as top pathways regulated by TAp73 (Table 4). Only 16 of these 153 target genes overlapped with the list of 343 validated p53 target genes [35]. These were *CDKN1A*, *CPEB4*, *DCP1B*, *FAS*, *FOSL1*, *GADD45A*, *GDF15*, *INPP1*, *MDM2*, *PANK1*, *PML*, *PRKAB2*, *RPS27L*, *RRAD*, *SERPINE1*, and *SFN*, and are known to have p53REs in their regulatory regions. 

We then compared the 81 validated target genes derived from individual studies with the 153 validated target genes from genome-wide studies and found 15 genes in common: *BCL2*, *BNIP3*, *FAS*, *GADD45*, *IVL*, *LIMA1*, *MDM2*, *CDKN1A*, *CDKN1C*, *PML*, *POSTN*, *GDF15*, *SERPINA1*, *SFN*, and *VEGF-A.* The signaling pathways that emerged from the analyses of these 15 overlapping genes included DNA damage response, cell cycle arrest, and apoptosis, similar to those obtained using the 81 TAp73 validated target genes. Among these, *BNIP3* is a pro-apoptotic protein that modulates the permeability of the mitochondrial membrane [44]. *CDKN1A* is a major target of p53 that induces cell cycle arrest upon DNA damage [123]. On the other hand, *POSTN* that codes for Periostin, activates the Akt- and FAK-mediated signaling pathways to promote cancer cell motility and the epithelial-mesenchymal transition [103]. 

Interestingly, a significant number of these targets regulated by TAp73 were also targets of other transcription factors, such as the AP-1 factors. For instance, treatment of head and neck cancer cells with the inflammatory cytokine TNF-α was found to lead to the re-distribution of TAp73 from p53RE sites onto AP-1 response element-containing sites [115], and was thereby suggested to support malignancy. Moreover, the co-occurrence of the AP-1 member c- Jun and TAp73 binding sites has been discovered, which allows both proteins to bind simultaneously and co-operate in target gene transactivation, often during growth promotion [112,113].

Taken together, the analyses of genome-wide TAp73 transcriptional targets indicate a varied role for TAp73 in cancer cell lines dependent on contextual settings, with both p53-like targets being identified as in the focused individual studies, as well as a larger number of genes regulated independent of the p53RE but in association with other transcription factors that are coupled to cellular survival and proliferation.

## 5. Non-Canonical Targets of TAp73 in Cancers

At the organismal level, p73 has been shown to be important for neuronal development, ciliogenesis, and reproduction, amongst others [37]. TAp73 regulates these functions through the activation of a myriad of target genes, and interestingly, many of these developmental targets have not been demonstrated to be regulated by p53 or to contain a typical p53RE [36,124,125]. These observations point to the fact that TAp73 can transactivate genes in a manner independent of canonical p53REs in their regulatory regions. 

Similar observations have been increasingly noted in studies using cancer cell lines, particularly in the context of cellular growth promotion. Most of these studies were initiated from the observation that an absence of TAp73 led to a reduction in cancer cell proliferation. Initial studies demonstrated that TAp73 is capable of augmenting the transactivation of AP-1 target genes such as *cyclinD1* and *collagenase* in a c-Jun-dependent manner, thereby promoting cellular growth [26]. Amelioration of TAp73 expression led to reduced growth, concomitant to reduced AP-1 target gene expression. Moreover, further studies showed that TAp73 could impede apoptosis by directly activating the transcription of IL1RAP and NEDD4L genes via a pathway involving c-Jun [112]. Interestingly, these anti-apoptotic target genes as well as many other TAp73-regulated genes were found to contain AP-1 binding sites in the proximity of the TAp73 binding regions in this study, suggesting the existence of a collaborative transcription factor network between TAp73 and AP-1 members. By contrast, the canonical pro-apoptotic target genes regulated by TAp73 were found to be devoid of the AP-1 binding sites in their promoter regions, suggesting that part of TAp73’s ability to promote cellular growth is dependent on the interplay between TAp73 and AP-1 members [24]. Consistently, stimulation of cancer cells with the inflammatory cytokine TNF-α was also found to re-distribute TAp73 onto promoters containing AP-1 response elements, as previously highlighted [115]. 

Other studies also found TAp73 to support cellular proliferation by enhancing cellular metabolism. TAp73 was shown to enhance the pentose phosphate pathway by directly activating the expression of *glucose-6-phosphate dehydrogenase* (*G6PD*) and promoted the Warburg effect via stimulating the expression of *phosphofructokinase 1 (PFKL),* both in human cancer cell lines as well as E1A+Ras-transformed mouse embryonic fibroblasts [27,28]. These TAp73 targets, similar to the AP-1 target genes regulated by TAp73, were not activated by p53, and were identified by candidate gene analysis based on the metabolic defects noted in TAp73-deficient cells. TAp73 was found to be bound to a site that is not entirely similar to the canonical p53RE, providing an explanation for the observed TAp73 specificity.

A role for the p73 proteins in hypoxia has also been well investigated. TAp73 was noted to inhibit angiogenesis by destabilizing HIF-1α via a protein–protein interaction, independent of its transcription activity, and thus indirectly led to the suppression of pro-angiogenic genes such as *Cxcl2*, *Ereg*, and *Vegf-c* [46,126]. On the other hand, TAp73 was also shown to promote angiogenesis, and thus tumor growth, by directly inducing a set of angiogenic genes independent of HIF-1α [29,45]. While seemingly opposite, these results likely represent a scenario in which TAp73 could act in both directions, depending on the context, thereby coordinating a feedback circuitry. Focusing on the transactivation properties of TAp73 in tumor promotion, some of the angiogenic target genes identified were found to have SP-1 binding sites in their promoter regions, suggesting that TAp73 could collaborate with other transcription factors to positively regulate vascular development in cancers [29,45]. Similar observations of TAp73 requiring SP-1 sites to activate cellular proliferation and anti-apoptotic genes such as *hTERT*, *CylcinB1*, and *Caspase-2S* have also been noted [23,72,85].

These data collectively demonstrate that TAp73 can have a distinct role in promoting cancer cell growth. TAp73’s ability to activate a unique and ever-expanding universe of transcriptional targets that do not converge with the canonical p53-like targets, often as a result of a collaborative co-existence with other transcription factors, highlight a more promiscuous function of TAp73 that is dependent on and dictated by the cellular contexts.

## 6. Discussion

Since its discovery over two decades ago, much effort has gone into deciphering the role of TAp73 in cellular growth control and in proving that TAp73 can be a substitute for p53, being able to perform similar functions through the transactivation of overlapping target genes. With this focused end goal in mind, many of the early studies were based on the “intended search and confirm” philosophy, thus unsurprisingly uncovering many common TAp73/p53 targets. However, both unbiased genome-wide studies and studies based on cellular phenotypes led to the revelation of many novel TAp73 targets that were associated with its role in cellular growth promotion, which is consistent with it being rarely mutated but over-expressed in human cancers.

**Figure 3:**
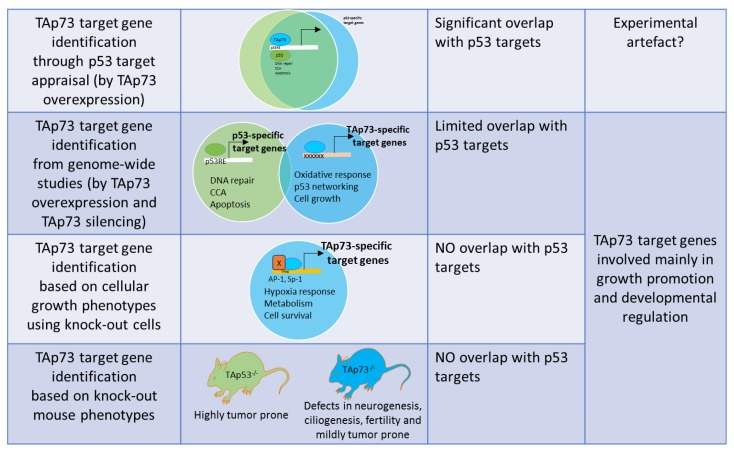
Overview of TAp73 target gene analyses from various studies, categorized based on the methodology broadly used for target gene identification. Venn-diagram schematically shows the extent of overlap between TAp73 versus p53 target gene. The bottom row describes the phenotypes of p53- or TAp73-null mice. p53RE: p53-response elements; TFRE: Transcription factor response element; xxxx: unknown binding sites; CCA: cell cycle arrest; X: Transcription factor.

The current analyses of TAp73 target genes collectively highlighted several points worth pondering (Figure 3). Firstly, while TAp73 has been clearly demonstrated to be able to transactivate p53 targets, whether this can occur in vivo needs to be re-evaluated. Most of the studies used overexpression systems and were performed in p53-null or mutant cells, to exclude confounding effects of p53. Though these studies have shown that TAp73 can bind to the target promoters containing p53RE under experimental conditions and lead to cell cycle arrest and apoptosis, p53-null cells do not generally utilize the often un-mutated endogenous TAp73 to enforce tumor suppression, or to activate p53 target genes by default. p53 loss has therefore been never compensated for by its family members. Yet, TAp73 has been suggested to contribute to cell death in many scenarios in the absence of p53, although this role appears to be secondary and observable at supra-physiological and highly intense experimental DNA-damage-inducing conditions. Loss of TAp73 in mice does however lead to spontaneous tumor development, albeit much later in life [31]. Similarly, co-heterozygosity of p53 and TAp73 leads to enhanced tumor development compared to p53^+/-^ mice [127,128], together indicating that while TAp73 has a role in tumor suppression, it is likely to be very minor and secondary. This further raises the provocative possibility that TAp73 was not evolved for tumor suppression, and regulation of p53 targets may not be its indigenous function.

Next, based on the primary observation that TAp73 is often overexpressed without being mutated in human cancers, a series of studies have now demonstrated that TAp73 expression can indeed be induced by growth-inducing conditions. Consequently, TAp73 has been suggested to be able to promote cellular growth and survival, mainly through the activation of target genes that appear to be context-dependent, and hence non-canonical. Importantly, it is emerging that these novel genes are often transactivated by TAp73 in collaboration with other transcription factors that promote cellular survival in various ways. These observations, together with the fact that many TAp73 target genes that regulate the developmental phenotypes noted in the absence of p73/TAp73 do not have a typical p53RE, indicate the promiscuity of TAp73 in being able to be adept in converging contextual signals to enable cellular survival. 

An interesting and yet often overlooked feature of these studies highlighting a pro-growth role for TAp73 is that most of the experiments were initially performed in MEFs that have been transformed, from which the target genes were identified and confirmed in human cancer cell lines. Importantly, these MEFs contained wild-type p53. Hence, it is tempting to speculate that in normal contexts where p53 is present, the primary role of TAp73 is context-dependent, being involved in regulating developmental genes at the early stages and for cellular proliferation and survival thereafter. Moreover, as a target of the key cell cycle regulator E2F-1 [129], it is not unimaginable that TAp73 has properties that will, when necessary, enable it to support cellular growth. This scenario appears to have been hijacked in tumors where TAp73 expression is elevated. TAp73 likely unleashes its survival instincts in cancer cells by co-operating with other transcription factors.

In summary, we conclude that TAp73, though being a structural homolog of p53, is not the latter’s natural functional substitute. In its own right, TAp73 has evolved to perform unique functions in regulating developmental processes and cellular growth, through entirely different mechanisms and through different sets of target genes that are not common to and cannot be substituted by p53. While TAp73 can perform p53’s functions, the opposite is not true. Thus, the p53 target genes identified to be regulated by TAp73 may indeed represent a virtual reality. 

## Figures and Tables

**Table 1:** List of genes validated as TAp73 direct targets from 71 focused studies.

Gene Name	No. of Studies Validated in	Description	Reference
ADA	2	Adenosine deaminase	[25,49]
ADAM17	1	ADAM metallopeptidase domain 17	[50]
AQP2	1	Aquaporin 2	[51]
AQP3	1	Aquaporin 3	[52]
Bak1	1	BCL2 antagonist/killer 1	[53]
Bax	12	BCL2 associated X	[42,54,55,56,57,58,59,60,61,62,63,64]
BCL2	1	BCL2 apoptosis regulator	[64]
BCL2L11	1	BCL2 like 11	[65]
BNIP3	1	BCL2 interacting protein 3	[44]
BDKRB2	2	Bradykinin receptor B2	[51]
S1002A	1	S100 calcium binding protein A2	[66]
CASP1	1	Caspase-1	[67]
CCND3	2	Cyclin D3	[25,54]
FAS	1	Fas cell surface death receptor	[61]
CDC2	1	CD2 molecule	[25]
CDC25C	2	Cell division cycle 25C	[25,68]
CDK1	2	Cyclin dependent kinase 1	[69]
CKS2	1	CDC28 protein kinase regulatory subunit 2	[70]
MMP-1	2	Matrix metallopeptidase 1	[24,26]
Cox4i1	1	Cytochrome c oxidase subunit 4	[71]
CCNB1	1	Cyclin B1	[72]
CCND1	2	Cyclin D1	[24,26]
CCNG1	1	Cyclin G1	[61]
DNAI1	1	Dynein axonemal intermediate chain 1	[73]
DNALI1	1	Dynein axonemal light intermediate chain 1	[73]
DNp73	2	Delta p73	[58,74]
FASN	1	Fatty acid synthase	[25]
FGFR3	1	Fibroblast growth factor receptor 3	[75]
FOXJ1	1	Forkhead box J1	[73]
GADD45	3	Growth arrest and DNA damage inducible alpha	[59,76,77]
Gast	1	Gastrin	[78]
GATA1	1	GATA binding protein 1	[79]
GLS2	2	Glutaminase 2	[80,81]
HAGH	1	Hydroxyacylglutathione hydrolase	[82]
GRAMD4	1	GRAM domain containing 4	[41]
H2BC5	1	H2B clustered histone 5	[59]
H2BC11	1	H2B clustered histone 11	[59]
H3C3	1	H3 clustered histone 3	[59]
H4C11	1	H4 clustered histone 11	[59]
Hey2	1	hes related family bHLH with YRPW motif 2	[83]
HIV-LTR	1	Human immunodeficiency virus 1	[64]
HMG1	1	High mobility group box 1	[84]
TERT	1	Telomerase reverse transcriptase	[85]
IGFBP3	1	Insulin like growth factor binding protein 3	[54]
IGFIR	1	Insulin like growth factor 1 receptor	[86]
Il4ra	1	Interleukin 4 receptor, alpha	[87]
IVL	1	Involucrin	[88]
ITGB4	1	Integrin subunit beta 4	[89]
JAG2	1	Jagged canonical Notch ligand 2	[66]
CD82	1	CD82 molecule	[90]
LIG4	1	DNA ligase 4	[43]
Lima1	1	LIM domain and actin binding 1	[91]
Loricrin	1	loricrin cornified envelope precursor protein	[88]
SERPINB5	1	Serpin family B member 5	[92]
MDM2	12	MDM2 proto-oncogene	[50,54,55,56,57,58,61,71,76,93,94,95]
ABCB1	1	ATP binding cassette subfamily B member 1	[95]
MIR3158	1	microRNA 3158	[96]
MIR34A	1	microRNA 34a	[96]
MMP13	1	Matrix metallopeptidase 13	[50]
NCAM	1	Neural cell adhesion molecule 1	[88]
PMAIP1	2	Phorbol-12-myristate-13-acetate-induced protein 1	[47,97]
CDKN1A	19	Cyclin dependent kinase inhibitor 1A	[40,47,50,56,57,58,61,64,66,68,71,76,78,84,88,89,98,99,100]
TP53AIP1	2	Tumor protein p53 regulated apoptosis inducing protein 1	[40,42]
CDKN1C	1	Cyclin dependent kinase inhibitor 1C	[101]
PDGFRB	2	Platelet derived growth factor receptor beta	[84,102]
PFKL	1	Phosphofructokinase, liver type	[27]
PML	1	Promyelocytic leukemia	[42]
POLD2	1	DNA polymerase delta 2, accessory subunit	[24]
POSTN	2	Periostin	[103,104]
GDF15	1	Growth differentiation factor 15	[66]
BBC3	3	BCL2 binding component 3	[47,62,105]
RAD51	1	RAD51 recombinase	[43]
RAD52	1	RAD52 homolog, DNA repair protein	[43]
SERPINA1	1	Serpin family A member 1	[66]
SFN	1	Stratifin	[66]
TP53INP1	1	Tumor protein p53 inducible nuclear protein 1	[106]
VDR	1	vitamin D receptor	[107]
VEGFA	1	vascular endothelial growth factor A	[108]
YBX1	1	Y-box binding protein 1	[99]
AFP	1	Alpha fetoprotein	[109]
Δ133p53α	1	TP53	[110]

**Table 2:** Signaling pathways deduced from TAp73 target genes identified from the focused studies. Top signaling pathways analyzed using the 81 TAp73-regulated target genes from the 71 individual focused studies were derived by analysis with Enrichr (https://amp.pharm.mssm.edu/Enrichr/).

Index	Name	*p*-Value	Adjusted *p*-Value	Odds Ratio	Combined Score
1	p73 transcription factor network	2.362 × 10^−37^	3.567 × 10^−34^	70.15	5916.51
2	MicroRNA regulation of DNA damage response	3.710 × 10^−26^	1.867 × 10^−23^	58.52	3426.69
3	TAp63 pathway	5.385 × 10^−22^	1.626 × 10^−19^	61.34	3003.83
4	p53 signalling pathway	4.478 × 10^−31^	3.381 × 10^−28^	39.87	2786.28
5	Inactivation of BCL-2 by BH3-only proteins	9.558 × 10^−9^	1.203 × 10^−6^	137.69	2542.64
6	Chk1/Chk2(Cds1)-mediated inactivation of cyclin B-Cdk1 complex	6.850 × 10^−7^	4.926 × 10^−5^	144.58	2052.12
7	p53 activity regulation	1.057 × 10^−23^	3.989 × 10^−21^	36.76	1944.61
8	TP53 network	1.104 × 10^−10^	2.382 × 10^−8^	76.09	1744.58
9	Cell cycle: G2/M checkpoint	1.173 × 10^−9^	1.969 × 10^−7^	53.55	1101.12
10	G2/M DNA damage checkpoint	5.686 × 10^−6^	2.960 × 10^−4^	80.32	970.09

**Table 3:** Overview of high-throughput studies for the identification of TAp73 target genes. KD: knockdown; OE: overexpression, WT: wild-type; CDDP: Cisplatin.

Cell lines (p73 Expression Regulated by)	p53 Status	Conditions	Techniques	Validated Genes	Reference
HCT116 (Control vs p73 KD)	WT p53	CDDP treated	Microarray	PML	[42]
H1299 (Control vs TAp73 OE)	Null p53, high TAp73	Normal growth condition	Microarray	No validation described	[120]
SK-Mel-29 (Control vs DNp73 OE)	WT p53	Normal growth condition	Microarray	LIMA1	[91]
H1299 (Control vs TAp73 KD)	Null p53 high TAp73	Xenografts in nude mice	Microarray	BNIP3, CITED2, FLH2, HSP40, LOX, P4HA1, PDK1, PLOD, SEPT9, VEGF-A, Zyx	[45]
U251 (Control vs p73 KD)	Mutant p53	72 hr post of transfection	Microarray	ANAX13, BCL2, CTGF, EDNRB, FLNB, ITGAX, LEFTY2, MAL, PDE4D, POSTN, RSG4, THSB4	[103]
HEK293T (Control vs p73 KD)	WT p53 inactivated by SV40	3 hr post of treatment with H_2_O_2_	Microarray	No validation described	[119]
H1299 (Control vs TAp73 OE)	Null p53, high TAp73	5 hr post of transfection	Microarray & ChIP-Seq (Antibody not specified)	CDKN1A, ACTN4, AXL, CREB3, CTNNBIP1, DDEF2, DLGAP1, FLJ35934, ITGA6, LTBP2, mdm2, MIG6, MOBKL2A, PANK1, RPS27L, RRAD, SVIL	[114]
Saos2 (TAp73α vs TAp73β OE)	Null p53, Null p73	Induced by doxycycline	RNA-Seq & ChIP-Seq (Antibody: BL906, Abcam)	BGN, CDK6, CDKN1A, CDKN1C, DCP1B, DEDD, FAS, GDF15, GHRL3, IL1RAP, Mdm2, METT10D, NDUFS2, NEDD4L, RNF43, SFN	[112]
HCT116-3(6)	HCT116 with altered MLH1	16 hr post of treatment with hydroxyurea	ChIP-array (polyclonal antibody: #827, home-made)	AARS, APOC3, ARRDC3, BAZ1A, BPIL1, C11orf21, C14orf169, C15orf21, C21orf94, CHRNG, CNFN, CNGB3, CSAG1, CX3CL1, DGPCR, DKFZp313M07, DKFZp566F0947, ETF1, FIGLA, FLJ45121, FLJ90637, FUT9, GDF1, GDF10, GMNN, GPR132, GPRASP2, GZMA, HAMP, HAPLN3, INPP1, IVL, KIAA0473, KIAA1404, KLF1, KLPH, LHX6, MAP1A, MEFV, MGC12458, MSN, NEURL, NKX28, PKDREJ, POLK, PP5423, RTN4RL2, SDS, SLCO5A1, SPR, STAM, STMN4, TWIST1, UBE2E1, ZNF157	[118]
Rh30 (shGFP vs shp73)	Mutant p53	Rapamycin	Microarray & Chip-array (Antibody: BL906, Abcam)	A2BP1, ACCN1, C20orf39, CLSTN2, CPEB4, EIF5AL1, FAM21B, GAS1, KCNMA1, MCEE, MDM2, NCK2, NOS1AP, NTN1, PDE6D, PRKAB2, PTCHD1, PTPRO, RPS27L, RRAD, TIAM2	[113]
ME180	WT p53 inactivated by HPV	Normal growth condition	ChIP-array (Antiboyd: 11C12, homemade)	No validation described	[121]
UM-SCC46	Mutant p53	1 hr post of treatment with TNF-α	ChIP-seq (Antibody: NBP2-24737, Novus Biologicals)	BCL3, CDKN1A, CEBPA, FOSL1, GADD45A, HBEGF, SERPINE1, TNFSF10	[117]

**Table 4:** Signaling pathways deduced from TAp73 target genes identified in the genome-wide studies. The 153 TAp73 target genes identified and validated in high-throughput studies were used to derive the Gene Ontology (GO) terms, using Enrichr (https://amp.pharm.mssm.edu/Enrichr/).

Index	Name	*p*-Value	Adjusted *p*-Value	Odds Ratio	Combined Score
1	TP53 network	2.451 × 10^−7^	1.157 × 10^−4^	34.86	530.56
2	Hypothesized Pathways in Pathogenesis of Cardiovascular Disease	3.491 × 10^−5^	2.746 × 10^−3^	21.19	217.49
3	Photodynamic therapy-induced AP-1 survival signalling	2.025 × 10^−6^	4.780 × 10^−4^	15.89	208.37
4	G1 to S cell cycle control	8.761 × 10^−6^	1.378 × 10^−3^	12.42	144.60
5	DNA damage response	1.248 × 10^−5^	1.472 × 10^−3^	11.69	131.96
6	miRNA regulation of DNA damage response	1.602 × 10^−5^	1.513 × 10^−3^	11.19	123.59
7	Oxidative damage	2.310 × 10^−4^	9.910 × 10^−3^	13.25	110.90
8	miRNAs involved in DNA damage response	5.574 × 10^−3^	0.1144	17.66	91.65
9	Non-small cell lung cancer	1.416 × 10^−4^	7.426 × 10^−3^	10.03	88.93
10	miR-517 relationship with ARCN1 and USP1	3.719 × 10^−2^	0.3735	26.49	87.20

## Supplementary Materials

The following are available online at https://www.mdpi.com/1422-0067/21/4/1346/s1. Supplementary Table S1: Detailed analyses of TAp73 target genes identified from 77 focused studies. Supplementary Table S2: Detailed analyses of TAp73 target genes identified from genome-wide studies.
